# Lifelong Exposure to Multilingualism: New Evidence to Support Cognitive Reserve Hypothesis

**DOI:** 10.1371/journal.pone.0062030

**Published:** 2013-04-30

**Authors:** Magali Perquin, Michel Vaillant, Anne-Marie Schuller, Jessica Pastore, Jean-François Dartigues, Marie-Lise Lair, Nico Diederich, on behalf of the MemoVie Group

**Affiliations:** 1 Centre for Health Studies, Centre de Recherche Public-Santé (CRP-Santé), Strassen, Luxembourg; 2 Methodology and Statistics Unit, CRP-Santé, Strassen, Luxembourg; 3 CRP-Santé now Unit of Educational Measurement and Applied Cognitive Science University of Luxembourg, Luxembourg, Luxembourg; 4 Cognitive Neurorehabilitation and Psychology Unit, Rehazenter, Luxembourg, Luxembourg; 5 Inserm U897 Université de Bordeaux II, Bordeaux, France; 6 Department of Neurosciences, Centre Hospitalier de Luxembourg (CHL), Luxembourg, Luxembourg; University of São Paulo, Brazil

## Abstract

**Objective:**

Investigate the protective effect of multilingualism on cognition in seniors.

**Methods:**

As part of the MemoVie study conducted on 232 non-demented volunteers aged 65 and more, neurogeriatric and neuropsychological evaluations were performed. Participants were classified as presenting either cognitive impairment without dementia (CIND) or being free of any cognitive impairment (CIND-free). Language practices, socio-demographic data and lifestyle habits were recorded. In this retrospective nested case-control design, we used as proxies of multilingualism: number of languages practiced, age of acquisition and duration of practice, emphasizing the temporal pattern of acquisition, and the resulting practice of several languages sequentially or concomitantly during various periods of life. This special angle on the matter offered to our work a dimension particularly original and innovative.

**Results:**

44 subjects (19%) had CIND, the others were cognitively normal. All practiced from 2 to 7 languages. When compared with bilinguals, participants who practiced more than 2 languages presented a lower risk of CIND, after adjustment for education and age (odds ratio (OR) = 0.30, 95% confidence limits (95%CL) = [0.10–0.92]). Progressing from 2 to 3 languages, instead of staying bilingual, was associated with a 7-fold protection against CIND (OR = 0.14, 95%CL = [0.04–0.45], p = 0.0010). A one year delay to reach multilingualism (3 languages practiced being the threshold) multiplied the risk of CIND by 1.022 (OR = 1.022, 95%CL = [1.01–1.04], p = 0.0044). Also noteworthy, just as for multilingualism, an impact of cognitively stimulating activities on the occurrence of CIND was found as well (OR = 0.979, 95%CL = [0.961–0.998], p = 0.033).

**Conclusion:**

The study did not show independence of multilingualism and CIND. Rather it seems to show a strong association toward a protection against CIND. Practicing multilingualism from early life on, and/or learning it at a fast pace is even more efficient. This protection might be related to the enhancement of cognitive reserve and brain plasticity, thereby preserving brain functions from alterations during aging.

## Introduction

Alzheimer's disease (AD) is a chronic and still incurable pathology. Synaptic loss invariantly occurs in early AD and its extent is strongly correlated with the severity of dementia. Altered synaptic plasticity and neuronal degeneration progressively lead to cognitive impairment and eventually AD. In the absence of appropriate treatment, attention has been focused on the preclinical stages of dementia, such as the so-called “cognitive impairment no dementia” phase (CIND) [1]. CIND is a clinical syndrome characterised by noticeable decline in memory or other cognitive abilities with little or no perceptible effect on daily functioning. Besides, the CIND syndrome does not meet the criteria for dementia listed in the Diagnostic and Statistical Manual of Mental Disorders, Fourth Edition, Text Revision (DSM-IV-TR) [2]. Beyond research on prodromal stages of dementia, there has also been a growing interest in risk factors for AD, as well as in factors that could protect against AD or delay its onset. Many authors have put forward evidence of how education [3]–[5], occupation [6]–[9], as well as leisure and social activities [10]–[12] requiring intellectual faculties increase cognitive reserve, and may thus delay cognition deterioration. Stern and Munn [13] advocated the necessity to closely explore the role of cognitive reserve in terms of intensity, frequency and cognitive components as required in activities of daily life. With its cosmopolitan environment and its three official languages, Luxembourg is a “natural laboratory of multilingualism”, where people have to switch permanently from one language to another. In this study, we explored the potentially protective effect of multilingualism against cognitive impairment in an elderly cohort. In line with the protection offered by lifelong bilingualism against AD onset, as reported by the group of Bialystok [14], we aimed to determine whether the practice of more than two languages would produce an even more potent effect. Furthermore, we also investigated whether the time of acquisition of several languages and the modalities of practice -sequential versus concomitant- have further effect. Finally, we looked into whether the linguistic effect can be clearly distinguished from the protective effect of other cognitively stimulating activities.

## Methods

Participants were recruited from the general population living in Luxembourg as part of the MemoVie prospective cohort study on cognitive aging and dementia [15]. Thereby, 232 volunteers without dementia completed cognitive and health screening questionnaires and provided socio-demographic data. All participants were questioned about their language practice. We estimated that with this sample size the study would have a power of 71%, assuming an α level of 5%, a proportion of individuals free of any cognitive impairment (CIND-free) practicing more than 2 languages of 94%, and an odds ratio (OR) of 0.33 of presenting CIND.

### Neuropsychological and clinical evaluation

A standardized in-person evaluation was carried out by trained psychologists, nurses as well as neurologists and geriatricians in face-to-face interviews. The choice of the location was left to the participants. The complete evaluation procedure and the neuropsychological as well as neurogeriatric assessment have been described in details [15]. Briefly, through a two-level process of classification, relying on a precise and thorough decisional algorithm, standardised and validated by an external expertise, the diagnoses were finally established as follows “absence of cognitive impairment”, “isolated cognitive complaint”, “cognitive impairment without cognitive complaint”, “mild cognitive impairment”, “dementia”, and “other cognitive impairment”. After excluding subjects with the last two diagnoses for the purposes of this study, participants were grouped into “free of CIND” and presenting “CIND”.

### Multilingualism evaluation

The multilingual ability of the participants was quantified through the number of fluent languages practiced all life long but also through the maximum of languages practiced concomitantly, the age at which each language was learnt, the status of practice (if still current at the time of the study), and the duration of practice (in years).

### Screening for leisure and socio-cultural features

Educational level was appraised, based upon the total years of formal and non-formal education. Subjects were questioned about their leisure and social life, as well as their physical activities. 29 different activities were proposed, and participants added 22 new activities to the list. The time typically spent performing these activities was recorded. Based on previous works [10], [16]–[18], we categorized leisure and socio-cultural activities into 5 components: social, cognitively stimulating, productive, recreational and passive, as well as physical component (non-sport). All components were graded as follows: 0 = none, 1 = low, 2 = moderate, 3 = high [16]. The involvement in each activity was rated on a 5-point scale: 5 points, every day or about every day; 4 points, several times a week; 3 points, several times a month; 2 points, several times a year; and 1 point, once a year or less [18]. The sum was calculated for each activity in the same component, weighted by the coefficient of involvement (0 to 5). Besides, sport (or strenuous activity) was alternatively explored with its frequency of practice.

### Statistics

The statistical exploration of multilingualism was conducted in two ways. Firstly subjects were analysed through two groups: a) bilinguals (subjects practicing 2 languages) and b) multilinguals (more than 2 languages). Secondly, in a separate analysis we considered subjects practicing 2, 3, 4 or more than 4 languages. Descriptive statistics based on means and standard deviations, numbers and percentages, OR and 95% confidence limits (CL) were used for the occurrence of CIND, multilingualism as well as risks factors.

Univariate analysis (Chi 2, Student t-test and Mann-Whitney test as appropriate) was used to explore relationships between either occurrence of CIND or multilingualism subgroups and: age of participants, education (duration of studies), gender, leisure and socio-cultural activities categories as well as number of languages practiced (for CIND exclusively).

All risk factors (leisure and socio-cultural activities) with a p-value below 0.05 were included in a saturated model, and a stepwise descending model-building procedure was carried out [19]. The likelihood ratio test between nested models and the Bayesian information criteria were used to assess the significance of the removal of each variable from the model. The final model was refined by looking at the linearity of the continuous variables in the logit. Orthogonal polynomials were calculated and the model was re-estimated with a penalized likelihood to reduce differences in units of measurement. Interactions between variables in the final model were also tested for significance. The same process was used to model CIND based on (a) duration of concomitant practice of 2, 3, 4, 5, 6 or 7 languages, (b) characteristics of the learning process, i.e. stopped at a specific stage of multilingualism or progressed with the acquisition of a new language, the (n+1)^th^, and (c) age at which the (n+1)^th^ language was learnt. We also considered this model by (1) grouping duration of practice of 1 and 2 languages in a “duration of pre-multilingualism” and duration of practice of more than 2 languages in a “duration of multilingualism”, (2) creating an age at which multilingualism began and, (3) a status of multilingualism where participants stayed in a pre-multilingualism or reached a multilingualism status (3 or more language practiced).

Adjustment was made for age and education. Indeed, both variables exhibited significant links with the occurrence of cognitive impairment and/or multilingualism respectively (data not shown). This configuration typically sets out a confounding effect in which the confounding factors correlate with both the dependent and the independent variables. Therefore, the assessment of the risk related to the effects of multilingualism on cognitive impairment implied the necessity to provide control for education and age, in order to not misjudge the specific effect of multilingualism. Other potential confounding factors such as gender and investigator were discarded since no influence was evidenced. A mixed model with a normal logit mixture was used with the location of the interviews as random effect, since the evaluation process of the participants was clustered in two different sites [15]. Forty-six subjects (19.8%) did not answer the question about the time of acquisition for at least one of their admitted mastered languages. A missing data replacement method was consequently applied [20], [21].

Exploratory in nature, our statistical analysis has been therewith constructed in a hierarchical manner. Hence, we defined that results meeting the threshold criterion of a p-value <0.05, uncorrected for multiple comparisons, would be statistically significant. All tests were two-tailed. Statistical analyses were carried out with the statistical package SAS System version 9.2 (SAS Institute, Cary, North Carolina, USA).

### Ethics

An informed consent form was signed by all participants. The study was approved by the National Research Ethics Committee and authorized by the National Commission for Data Protection of Luxembourg.

## Results

### Characteristics and description of multilingualism

**Figure 1:**
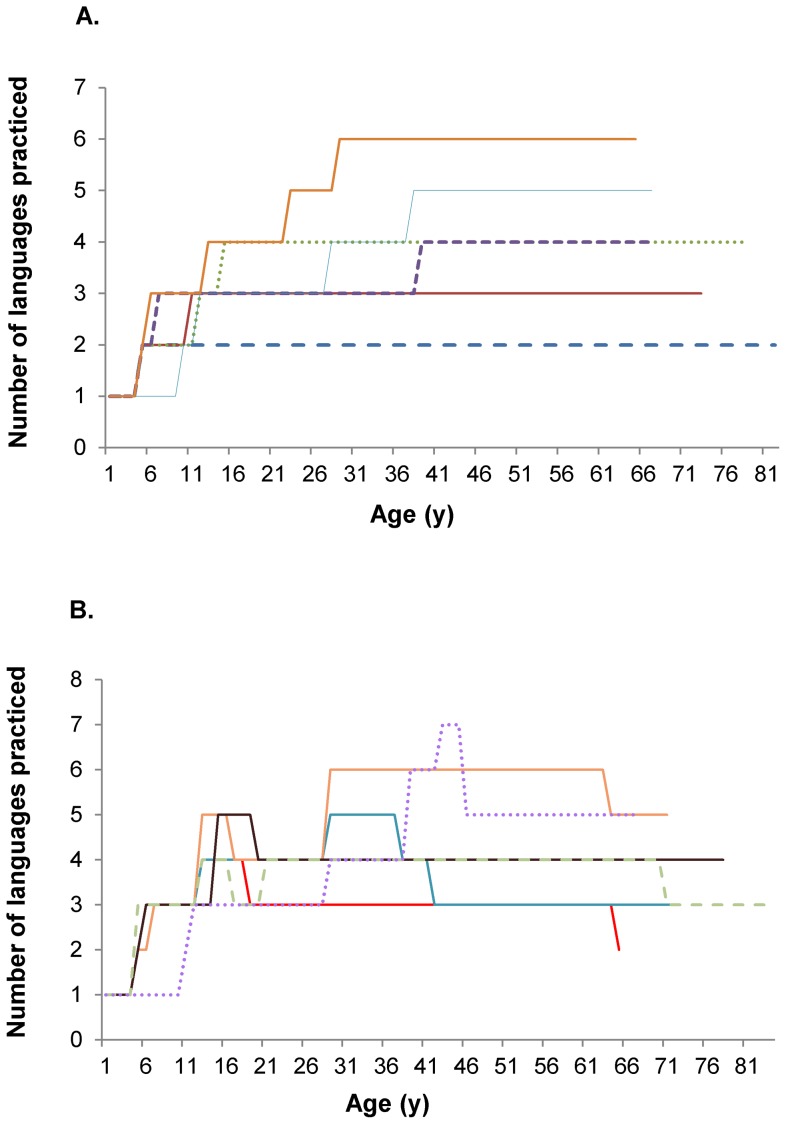
Acquisition of multilingualism in the studied population. Part A. Typical progression: sequential and increasing process of learning and practicing multilingualism. (For each stage of multilingualism, either individuals stayed at a specified level, or they progressed to the next step i.e. they learned an additional language.) Description of the 6 models of progression found among the 168 individuals for which the learning process represents the acquisition of one language after another (keeping the practice of all of them). Part B. Atypical and unordered progression of learning and practicing multilingualism. Illustration of the 6 models of atypical progression exhibited by the 64 subjects learning 2 or 3 languages in the same period of time and/or losing the practice of one or 2 languages at some point in their lives.

Participants have practiced from two to seven languages. So, of note, nobody remained monolingual throughout his life. Consequently, bilingual subjects were the reference group for all comparisons. The process of acquiring multilingualism was mainly achieved by learning languages one by one in a sequential process (for 168 out of 232 volunteers, 72.4%). The milestones of this typical learning process are represented in Figure 1A, where 168 observations are covered by 6 patterns. The remaining 64 individuals (27.6%) followed a different and atypical progression pattern: learning simultaneously several languages, and/or abandoning the practice of 1 or 2 learned languages at some point in their lives (Figure 1B).

### Characteristics of participants

Forty-four (19%) participants presented CIND, and 188 (81%) were CIND-free. Table 1 shows the main characteristics of the entire population (n = 232) and comparisons between CIND and CIND-free subjects. Subjects free of CIND had significantly higher education, number of languages used concomitantly or life through, and scores in the different components of socio-cultural and leisure activities (except productive component and sport). They were also significantly younger (2 years) than CIND participants. These analyses are descriptive in nature, and only the modelling strategy can establish associations.

**Table 1:** Characteristics of participants.

	Individuals with CIND (n = 44)	CIND-free individuals (n = 188)	Total population (n = 232)	*p*-value (CIND vs. CIND-free)
Female vs. male ratio	0.8	1.5	1.3	0.04^a^
Education, y	10.4 (3.7)	12.0 (3.6)	11.7 (3.6)	0.003^b^
Age, y	74.0 (5.3)	72.1 (5.1)	72.5 (5.2)	0.03^b^
Number of languages practiced life through	3.3 (0.9)	3.7 (1.0)	3.6 (1.0)	0.004^b^
Ratio:>2 vs. 2 languages practiced life through	4.5	19.9	12.6	0.006^d^
Maximum number of languages used concomitantly	3.2 (0.8)	3.7 (0.9)	3.6 (0.9)	0.002^b^
Ratio:>2 vs. 2 languages used concomitantly	4.5	17.8	11.9	0.009^d^
Social component, score	32.1 (13.7)	38.8 (16.5)	37.6 (16.2)	0.01^c^
Cognitively stimulating component, score	63.8 (21.6)	78.6 (21.3)	75.8 (22.1)	<0.0001^c^
Productive component, score	9.4 (6.6)	11.9 (7.7)	11.4 (7.5)	0.08^b^
Recreational and passive component, score	28.6 (7.5)	31.7 (7.3)	31.1 (7.4)	0.02^b^
Physical (non-sport) component, score	38.1 (14.4)	44.5 (13.3)	43.3 (13.7)	0.004^c^
Sport (or strenuous activity), min/week	159.5 (402.5)	236.9 (539.5)	222.2 (516.4)	0.09^b^

Data are given as mean (standard deviation) unless otherwise stated. a: Chi-square test; b: Mann-Whitney test; c: Student t-test; d: Fisher test. For each component, the total scores could potentially vary between 0 and the component specific value, indicating the highest contribution to the component: 0–280 for the social component, 0–467 for the cognitively stimulating component, 0–130 for the productive component, 0–76 for the recreational and passive component, 0–206 for the physical component (non-sport).

### Protection against CIND by multilingualism

**Figure 2:**
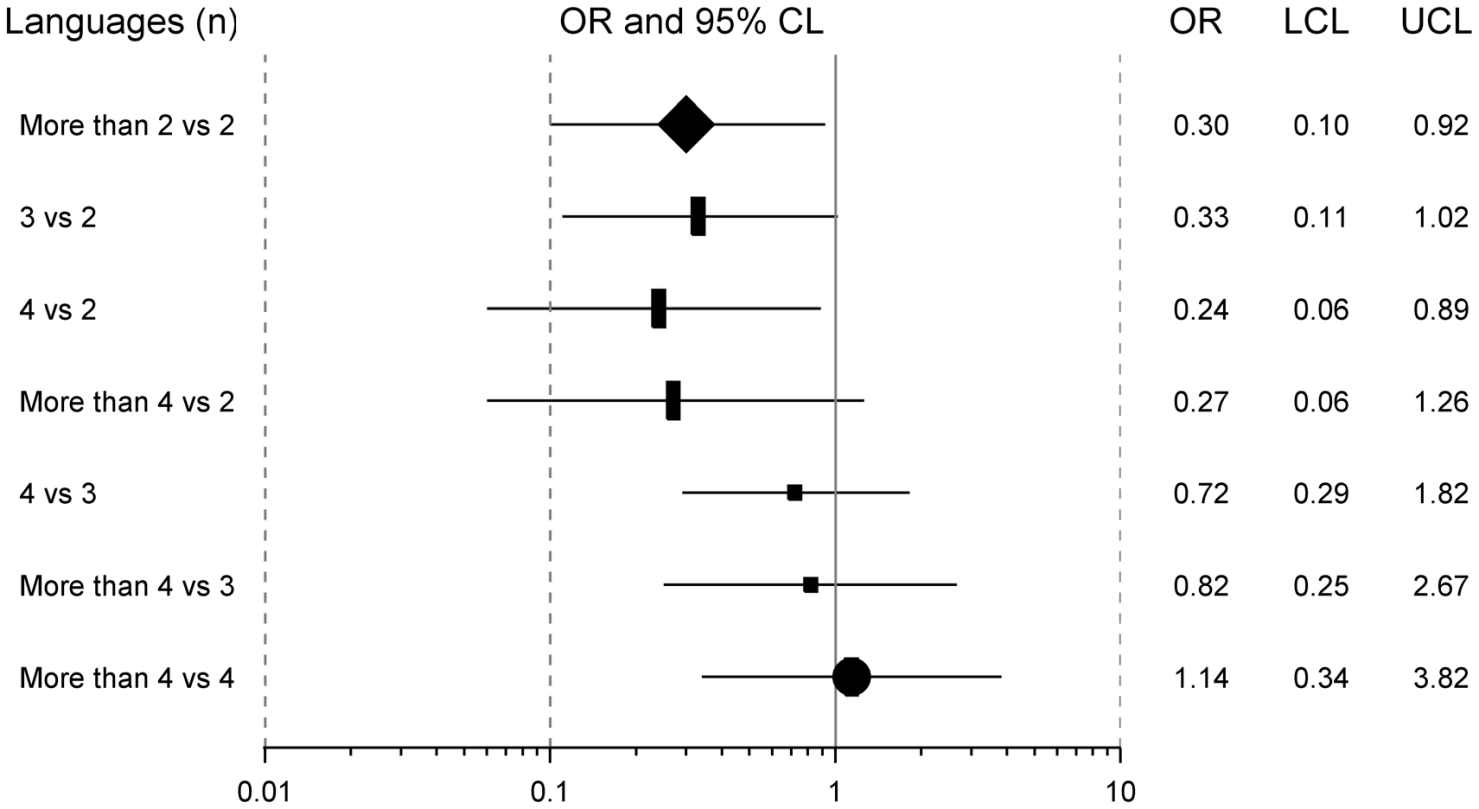
Association between different stages of multilingualism and CIND. Forest plot of the OR comparing different stages of multilingualism over lifetime, to bilingualism, trilingualism and/or quadrilingualism. The solid vertical line shows an OR of 1 (no effect). Each multilingual status is represented by a square, the size of which shows the corresponding sample size in the analysis. The symbols represent the four different analysis performed. The lozenge at the top shows the overall effect of speaking “more than two languages” versus “exactly two languages”. The CL for each situation is represented by a horizontal line and the lower and upper control limits are indicated next (LCL, UCL).

When compared with bilingual subjects, seniors who practiced through their life more than 2 languages were 3 times less likely to have CIND (OR = 0.30, 95%CL = [0.10–0.92]). A similar pattern was observed for 3 languages, and especially for 4 languages, the OR values decreasing from 0.30 to 0.24 (Figure 2). However, beyond this level of multilingualism, the situation becomes less clear, and *when compared with the trilingual subjects*, individuals who practiced 4 or more than 4 languages, presented the same probability of developing cognitive impairment. Similar results were observed when comparing participants practicing more than 4 languages with those practicing only 4. Comparable results were obtained considering the maximum number of languages practiced concomitantly (data not shown).

### The earlier and faster, the better

By modelling duration of pre-multilingualism (i.e. before practicing 3 languages), we observed that an increase of one unit (year) of the pre-multilingualism duration multiplied the risk of CIND by 1.022 (OR = 1.022, 95%CL = [1.01–1.04], p = 0.0044).

In addition, logistic modelling of CIND was conducted, involving the set of features depicted in Figure 1, such as duration and practice of n languages, characteristics of the learning process (i.e. stopping at a certain stage of multilingualism or progressing to a higher stage) and the age of learning of a new language. We obtained the evidence that in subjects directly progressing from 1 to 3 languages, the probability to be free of CIND is 13 times as high as that of the bilinguals (OR = 0.08, 95%CL = [0.01–0.86], p = 0.0373). Moreover, when the participants progressed from 2 to 3 languages, they are 7 times more prone to be protected against CIND than bilinguals who stayed at this level (OR = 0.14, 95%CL = [0.04–0.45], p = 0.0010).

### Other components of cognitive reserve as confounding factors?

Leisure and socio-cultural activities were found to be significantly lower in subjects with CIND in the univariate analysis (Table 1). They were therefore included in the multivariate analysis.

Following the descending modelling procedure (the final model explaining CIND), the cognitively stimulating component (CSC) on its own showed a protective effect quantified by an OR = 0.979, 95%CL = [0.961–0.998], p = 0.033 (Table 2). However, when multilingualism (through its 2 classifications mentioned in methods) and CSC were added to the model, neither was still significant (Table 2). Similar results were obtained after refinement of the model by using orthogonal polynomials and penalized likelihood. Also explored, interaction between both factors (multilingualism and CSC) did not show any effect (p = 0.6013), eliminating the possibility of a differential effect of multilingualism with high CSC. Based on these results, we concluded that despite the specific contributions of multilingualism and CSC to the occurrence of CIND, there did not seem to exist a prevailing protective effect on cognition of one factor over the other.

**Table 2:** Odds ratio for occurrence of CIND for practicing cognitively stimulating activities and multilingualism during lifespan.

Model terms	OR	95%CL	p-value
CSC	0.979	0.961–0.998	0.033
CSC	0.981	0.962–1.000	0.054
>2 vs 2^1^ languages	0.341	0.110–1.051	0.061
CSC	0.981	0.962–1.001	0.056
3 vs 2^2^ languages	0.366	0.116–1.149	0.085
4 vs 2 languages	0.270	0.071–1.027	0.055
≥5 vs 2 languages	0.329	0.069–1.556	0.160

Multilingualism was studied through its two classifications: ^1^subjects using 2 languages versus more than two; ^2^subjects using only 3, only 4 or more than four languages; CSC: cognitively stimulating component.

## Discussion

The MemoVie cohort findings, suggest a protective effect of actively-practiced multilingualism against the occurrence of CIND: this protection is even increased with earlier and more rapid acquisition of multilingualism. Moreover, the protection is especially visible at an even higher stage of multilingualism. Indeed, considering bilingual individuals as a reference, the probability of being later free of CIND increased up to 4 times along with the number of practiced languages life through. Two recent case reports of demented patients, allowed the authors to speculate on the persistent activation associated with multilingualism, explaining the slower rate of degeneration or functional inactivity observed (by neuropsychological tests and by SPECT images) in the multilingual patient compared with the bilingual one [22].

However, this unprecedented detected protective effect is probably limited by a “plateau” level as individuals who practice 4 languages or more have the same (reduced) probability of developing CIND as trilingual subjects. This trend was also observed in participants practicing more than 4 languages when compared with individuals practicing 4 languages only.

Based on the hypothesis that concomitant practice of several languages, which varies throughout life, produces a more significant effect on cognition than the overall (and lifelong) number of languages, the maximum number of languages used concomitantly was studied. However, our results did not show higher protection in the case of concomitance of practice than that of exposure to a certain number of languages during the entire life.

Further analysis explored the time needed by the subjects to become multilingual. It showed that the earlier individuals learn to speak more than 2 languages, the more pronounced the protective value. A clear positive trend actually appeared between the duration of the pre-multilingual period and the occurrence of CIND.

The study also confirms that subjects free of CIND systematically performed more leisure and sociocultural activities than subjects with CIND. They also had a higher level of education. These results are consistent with previous findings which demonstrated that the cerebral reserve capacity builds up on multiple factors such as education [8], [23], occupation [8], attainment and leisure activities [10], [13], [24].

A concern of this study pertained to the actual part of multilingualism which can be held accountable for the protection against CIND, out of all these various lifetime exposures. We therefore grappled with the issue of the other activities reported by subjects that may impact on cognitive reserve according to literature. Importantly, we found an impact of cognitively stimulating activities on the occurrence of CIND. However, our results did not determine a predominance of one of these factors but rather a similar effect leading one to conclude that multilingualism as well as other cognitively stimulating activities contribute to building up the cognitive reserve.

Our findings altogether corroborate those of Kavé et al. [25], which revealed in a non-demented Israeli elderly population, better cognitive performance as a function of the number of languages spoken (2, 3, or more), irrespective of education level. Our results furthermore complement those of Craik et al. [14], [26] and others [27], [28], who concluded that by contributing to cognitive reserve, lifelong bilingualism confers protection against AD onset. Chertkow et al.[29] emphasized a consistent protective effect in individuals speaking 3 or more languages, although they did not manage to properly replicate Craik's results on bilingual subjects [14] in a comparable Canadian population. They subsequently pointed out the complexity of the multiple influences that might contribute to cognitive reserve.

To the best of our knowledge, this study is the first one to associate the time dimension of language acquisition -earlier is better- with the later occurrence of CIND. This study however shows several limitations. First, the sample size is small. However, the power calculation showed that the study is sufficiently robust to establish our results. Moreover, the studied population reflects the multilingual environment of Luxembourg. Therefore, we were able to compare a differential multilingualism -from 2 to 7 languages- which consists of the main strength of this study. Even so, it could be argued that the absence of monolingual participants is a serious limitation of this study. However, monolinguals are extremely rare in Luxembourg and consigned to particularly low social and educational backgrounds. Extreme values of both variables would have been a considerable confounding factor. A monolingual controlled study would have been optimal. Nevertheless, the higher susceptibility to present neurodegenerative diseases in monolingual subjects than in bilingual ones has been shown by others [14]. This allowed us to consider the bilingual group as a reference in this work. We are aware of the lack of knowledge about the premorbid cognitive status of the studied population, especially due to the cross-sectional design of this study. However a poor a-priori functioning could have precluded the acquisition of several languages, or limited their practice. Furthermore, local education programs strongly encourage and facilitate languages acquisition, even in pupils with learning difficulties.

In addition, recall bias should not be excluded. Its extent is however limited, since the milestones of languages acquisition were mostly anchored in school education programs, making it easier for subjects to correctly recall the year of acquisition and the length of the learning phase. Finally, we did not consider the origin of the languages practiced to establish subgroups of people, or draw an objective assessment of the language skills reported. Despite these limitations and a subjective measure such as the number of practiced languages, we obtained important estimators of association with low variability.

Taken together, our results suggest that the cognition benefit provided by multilingualism peaks at 3 languages. This assumption was confirmed by the 7-fold difference, in terms of protection, detected in the case of multilingual subjects who progressed from the practice of 2 to 3 languages, compared with subjects who stayed permanently bilingual. In addition, the participants who simultaneously acquired 2 languages were even more favoured, with a 13 times higher protection as opposed to participants who did not learn more than 2 languages. Our results therefore seem to converge towards the notion of a cognition benefit from a threshold of 3 languages, practiced as early in life as possible. Multilingualism certainly contributes to providing greater reserve which is protective by delaying in time the clinical expression of dementia [30]. The reserve theory has been established through two main concepts, initially individualized, although most likely mingled. The first one named the “cognitive reserve”, also described as the “active reserve” implies physiological variability at the brain network level (synaptic organization, brain regions utilization); while the second concept, the so-called “brain reserve” or “passive model of reserve” implies differences in the quantity of available neural substrates [31] [7].

We could hypothesise that the “plateau” phenomenon emerging from our results, could reflect the critical threshold being described by authors [30], [31] in line with the brain reserve concept implying that, at some point, individual features (brain volume, neuronal and synaptic counts, dendritic branching), are likely to be altered by brain damage that could occur at the various ages of life. Thus this concept states that brain reserve should prove protective simply by creating distance from the cut-off threshold of functioning for dementia [30], [31].

Moreover, assuming that the multilingual ability occurs within a population exhibiting potentially higher inherent brain reserve, we could suppose that our study population is rather homogeneous in terms of brain reserve. Evidently the presupposition of this homogeneity could also corroborate the notion of “multilingualism plateau” which is no more protective above a common limit of 3 practiced languages.

On the other hand, it now clearly appears that cognitively stimulating experiences, associated with increased cognitive reserve (the concept of active model), have a direct effect on the brain [7], [8]. Therefore, brain reserve could presumably be maintained and even increased based on successful cognition-challenging experiences. This could simultaneously cover different aspects of memory-based activities, including learning and fluent practise of different languages as discussed in this paper. Supposing that “passive” brain reserve is homogeneously distributed in the entire population, the observed threshold of 3 languages could imply that at some point, the protective and compensatory processes inherent in “active” cognitive reserve have reached the maximum contribution that they can provide and cannot be further improved. To our knowledge, such a phenomenon of reserve threshold has never been described, and we infer that it can be noticeable only in populations exhibiting very high cognitive reserve levels. We do not however dismiss the possibility that reserve may continue to increase through other stimulating cognitive tasks calling on different cerebral structures or networks than those involved in language production.

Since cognitive reserve is also recognised as a factor influencing the rate of cognitive decline after diagnosis of AD (increasing the decline in case of high reserve) [24], the extent to which multilingualism can impact cognitive decline could be assessed through follow-up of the MemoVie cohort.

In addition to the neural mechanisms that underlie language development, social interactions can also be of benefit along with language acquisition, as pointed out by Kuhl's “social gating hypothesis” [32]. According to this hypothesis, increasing attention and/or arousal, information, sense of relationship are linked to the activation of brain mechanisms linking perception and action. Despite methodological differences, all these studies however conclude that the brain is malleable, with extraordinary adaptive capacities, and able to reorganize fast. Even in the absence of information about the level of proficiency, our findings robustly support the assumption that brain plasticity persists with age. This is especially favoured by continuing cerebral stimulation throughout life, which is obviously the case for our population. This may provide substantial benefits when facing neurodegenerative damage.

Further studies are needed to confirm these findings and determine whether the protection is limited to thinking skills related to language or if it also extends beyond that and benefits other areas of cognition.
